# Dentists and the R. C. of S.

**Published:** 1860-10

**Authors:** 


*Dentists and the R. C. of S.*—One hundred dentists have taken the License from the R. C. of S. of England, evidencing great want of co-operation in their dental school system.

When a faculty ceases to conduct a dental college upon a proper basis, the profession are justified in refusing to support them, but not otherwise. Money making should be lost sight of, and he who cannot, or will not, give his arduous labor without compensation, is not fit for so honorable a post.

A second, or any added occupation to that of the dentist, most properly disqualifies him for membership of any of the established Dental Societies of England. B.

